# The Persian Version of the “Life Satisfaction Scale”: Construct Validity and Test-Re-Test Reliability among Iranian Older Adults

**DOI:** 10.1007/s10823-017-9340-6

**Published:** 2017-12-12

**Authors:** Manije Moghadam, Mahyar Salavati, Robab Sahaf, Maryam Rassouli, Mojgan Moghadam, Ahmad Ali Akbari Kamrani

**Affiliations:** 1 Iranian Research Center on Aging, University of Social Welfare and Rehabilitation Sciences, Koodakyar Ave, Daneshjoo Blvd, Evin, Tehran, 1985713834 Iran; 2 Department of Physical Therapy, University of Social Welfare and Rehabilitation Sciences, Tehran, Iran; 3 Pediatric Nursing Department, Nursing & Midwifery School, Shahid Beheshti University of Medical Sciences, Tehran, Iran; 4 Department of Physiotherapy, School of Rehabilitation Sciences, Shahid Beheshti University of Medical Sciences, Tehran, Iran

**Keywords:** Life satisfaction, Life satisfaction scale, Older adults, Persian, Reliability, Validity

## Abstract

After forward-backward translation, the LSS was administered to 334 Persian speaking, cognitively healthy elderly aged 60 years and over recruited through convenience sampling. To analyze the validity of the model’s constructs and the relationships between the constructs, a confirmatory factor analysis followed by PLS analysis was performed. The Construct validity was further investigated by calculating the correlations between the LSS and the “Short Form Health Survey” (SF-36) subscales measuring similar and dissimilar constructs. The LSS was re-administered to 50 participants a month later to assess the reliability. For the eight-factor model of the life satisfaction construct, adequate goodness of fit between the hypothesized model and the model derived from the sample data was attained (positive and statistically significant beta coefficients, good R-squares and acceptable GoF). Construct validity was supported by convergent and discriminant validity, and correlations between the LSS and SF-36 subscales. Minimum Intraclass Correlation Coefficient level of 0.60 was exceeded by all subscales. Minimum level of reliability indices (Cronbach’s α, composite reliability and indicator reliability) was exceeded by all subscales. The Persian-version of the Life Satisfaction Scale is a reliable and valid instrument, with psychometric properties which are consistent with the original version.

## Introduction

The world’s global population is graying. The UN Population Division projected the number of people over age 60 would increase from just under 800 million in 2011 to just over 2 billion in 2050 (Bloom et al. 2011). The aging population in developing countries is estimated to increase more than 2.5 times, compared with a 71% increase in developed countries (Lux and Scherger 2017). According to last census of Iran population conducted in 2011, about 8.23% of people in Iran aged 60 years and over (Statistical Center of Iran 2011). As the trend in many countries around the world, Iran started to experience graying of population as well (Shirazikhah et al. 2012; Amini et al. 2013) and there will be a rise in the rate of growth of the elderly population after 2015, so that its size will double between 2015 and 2030 (Mehryar and Ahmad-Nia 2004).

The increased life expectancy means that people have an opportunity to live a longer life. However, maintaining the quality of life in these extended years and its subjective proxy measure namely life satisfaction would be a major concern in the elderly population as well for gerontological research.

Life satisfaction has been defined as “a global evaluation by the person of his or her life”(Pavot et al. 1991). It has been described as the subjective expression of quality of life (Fernandez-Ballesteros et al. 2001).The psychological well-being and life satisfaction, as its fundamental component, is of great importance at older ages (Parker et al. 2008).

Life-satisfaction has also been used to evaluate the effects of social policies particularly those that aim to improve the quality of life. It can be used as an outcome measure for assessment of the interventions (Veenhoven 1996).

Life satisfaction is a broad concept and has been measured by using different instruments. One of the instruments that measure life satisfaction specifically in older adults, considering the entire domain of this concept, is the “Life Satisfaction Scale (LSS)”. The LSS is a revised version of “Life Satisfaction in the Elderly Scale” developed by Salamon and Conte in 1984. This is a longstanding measure of life satisfaction with optimum psychometric properties (Salamon and Conte 2003). The LSS has been proved to be convenient and user-friendly to administer. Studies of psychometric properties of LSS indicate that it is an appropriate instrument, both clinically and theoretically (Salamon 1988).

Despite the importance of the concept of life satisfaction in the elderly, there is no valid and reliable scale in Persian language regarding this concept. Therefore, the present study was conducted to translate the original version of the LSS into Persian and to evaluate the psychometric properties of the Persian version of this scale in Iranian older adults.

## Materials and Methods

### Procedure

The present study is a methodological research through which the life satisfaction scale has been translated into Persian and the psychometric properties of the Persian version of LSS has been evaluated.

The LSS, a background variables questionnaire including demographics (ages, sex and educational level), and the Persian version of the Short Form Health Survey (SF-36) (Montazeri et al. 2005) as a questionnaire of a related construct were administered to 334 volunteer Persian speaking elderly people living in Tehran, Iran. The participants were selected from general public using convenience sampling based on inclusion criteria. They were community-dwelling older adults who were members of the elderly centers of Tehran municipality, and members of the elderly retirement centers. The appropriate sample size was determined based on Sample Size for Factor Analysis Method that suggested 300 provides a moderately good quality for factor analysis (Comrey and Lee 1992). Among the total number of 450 survey packets that were successfully delivered, 400 were returned. 66 questionnaires were considered invalid because more than one item in a subscale were missing or the overall number of missing items was more than 20%. In addition, 70 older adults agreed to participate in the test-retest reliability study. Among them, we selected 50 people who didn’t report any recent unexpected event that might influence their judgement about their own life satisfaction. To assess the test-retest reliability the questionnaire was re-administered to 50 participants (20 female, and 30 male) a month later. The eligibility criteria for participation in this study were adults with 60 years of age and older, literacy (at least 9 years of school education), and normal cognitive function (based on the results of clock drawing test as a cognitive function screening tool) (Aprahamian et al. 2009). After selecting eligible elderly and explaining the general nature of the study, the participants who signed the consent form for participation completed the LSS and SF-36 as self- report questionnaires. Furthermore, a single question with 5 point Likert-type scale responses concerning the overall satisfaction with life was asked. The completion of the questionnaire took about 20–25 min.

### Translation Process

After obtaining permission from the developers of the LSS, the scale was translated from its original source in English into Persian, according to the International Quality of Life Assessment (IQOLA) project guidelines (Bullinger et al. 1998).

Two translators who were native speakers of Persian, translated the LSS into Persian according to the standard IQOLA protocol. The two translators working independently, produced two preliminary translations of the LSS. Next, together with the researchers, the translators compared individual translations and then agreed on a common translation. Then, another native Persian translator evaluated the quality of the common translation, considering factors including clarity, common language use, conceptual equivalence and acceptability and modified the translation of items, if it was necessary (Bullinger et al. 1998).

Finally, an American bilingual translator translated the forward translation back into English to produce the backward version. The conceptual equivalence of the backward translation to the original version was verified by the developers. Then during the cognitive debriefing interview, 20 older adults were asked to complete the questionnaire to find any obscure, confusing and difficult items (Bullinger et al. 1998).

### The Instruments

#### Life Satisfaction Scale

The LSS is a multi-factor questionnaire which has been developed by Salamon and Conte to assess the life satisfaction. The LSS was developed primarily as a scale to measure life satisfaction in older adults by considering the whole domain of life satisfaction indicators. The LSS consists of eight subscales derived from 40 items with a five points Likert type scale responses. The eight subscales of the LSS include: daily Activities, meaning, goals, mood, self-concept (positive self-concept), health, finances, and social contacts (Salamon and Conte 2003). Each subscale of LSS consists of five items. The items of LSS were designed in a summative Likert format with a five point range. Therefore, the total score of each subscale may range from 5 to 25. For the complete LSS, total scores can range from 40 to 200 (Salamon 1988).

### The Short Form (36) Health Survey (SF-36)

SF-36 is a 36-item, self-administered measure of health-related quality of life. It consists of eight subscales including physical functioning, bodily pain, role limitations due to physical health problems, role limitations due to emotional problems, emotional well-being, social functioning, energy/fatigue, and general health perceptions. Scores for each subscale range from 0 (poor health status) to 100 (good health status) (Ware Jr and Sherbourne 1992). The Persian version of SF-36 has been previously validated in Iran (Montazeri et al. 2005).

### Clock Drawing Test

The Clock Drawing Test (CDT) is a simple neuropsychometric measure that can be easily administered to assess cognitive functions especially in elderly (Cahn et al. 1996; Brodaty and Moore 1997). The CDT score provides reasonably good screening test for dementia (Aprahamian et al. 2009). In a standard way of the clock drawing test the person is asked to draw the face of a clock, put all the numbers in correct positions, and set the hands at “10 after 11” (Hubbard et al. 2008). There are two general clock drawing test scoring approaches, including qualitative and quantitative scoring systems (Nair et al. 2010).

### Data Analysis

The structural equation modeling (SEM) is a powerful method for validating a measurement in the field of psychology (Yu and Hsu 2012). In this statistical methodology, structural theory bearing on some phenomenon, is analyzed by taking a confirmatory approach (Byrne 2001). Confirmatory factor analysis (CFA) is a technique of structural equation modeling which is used to assess the goodness of fit between a hypothesized model and the data gathered from the study samples (Kline 2011).

The partial least squares approach to SEM (or PLS path modeling) is a powerful data analytical tool applied in different fields of research.The PLS could be used as a first step to find the existing relations and to analyze the real world data (Rosipal and Krämer 2006). PLS can simultaneously test the structural model (relationships between constructs) and the measurement model (relationships between indicators and their corresponding constructs) to ascertain the overall psychometric properties of the scales, and the important relationships among the variables (Barclay et al. 1995; Hulland and Business 1999). PLS path modeling has the advantage of not assuming the normality of data distribution (Bagozzi 1994a, b).

In the present study, a confirmatory factor analysis followed by PLS analysis was performed, using the software SmartPLS2.0, to analyze the validity of the model’s constructs and the relationships between the constructs. Construct validity evaluated through the presence of two important aspects of measurement model; convergent and discriminant validity (Gefen et al. 2000).

Convergent validity can be evaluated by assessing indicator reliability, composite reliability, and average variance extracted (Fornell 1982).

Item’s factor loadings for their respective construct were calculated to assess indicators reliability (Fornell and Larcker 1981). The internal consistency reliability is indexed by the composite reliability. The recommended threshold value of 0.7 was considered (Nunnally et al. 1967). Cronbach’s alpha also was measured to assess internal consistency. Cronbach’s alpha values between 0.70 and 0.95 are considered satisfactory for internal consistency (Terwee et al. 2007).

The average variance extracted (AVE) represents the amount of variance a construct captures via its items relative to the amount of variation due to measurement error. According to the guidelines of Fornell and Larcker, if the AVE is less than .50, the validity of the construct is questionable (Fornell and Larcker 1981).

The discriminant validity determined through comparison of item loadings with item cross loadings. Each item should load more highly on its respective constructs than on other constructs (Gefen and Straub 2005).

Furthermore, to establish discriminant validity, Fornell and Larcker (1981) suggest that the square root of AVE in each latent variable should be greater than other correlation values among the latent variables (Fornell and Larcker 1981).

The structural model represents the relationships between constructs that were hypothesized in the model. The overall model goodness of fit determined through paths (statistical and practical significance) and coefficients of determination (R-square) (Gil-Garcia 2008). Also, a global criterion of goodness-of-fit (GoF) calculated as an index for validating the PLS model globally (Tenenhaus et al. 2005). For global validations of a PLS model, Wetzels, Odekerken-Schroder, and van Oppen have formulated indicative GoF values as baseline values: GoFsmall = .1, GoFmedium = .25, and GoFlarge = .36 (Wetzels et al. 2009).

In addition, a priori hypothesized patterns of associations between a scale and other validated instruments provide further evidence for the scale construct validity. It was hypothesized that the correlations between the LSS mood scores and SF-36 emotional wellbeing scores should be high; also, the correlations between the LSS health scores and SF-36 general health perception scores should be high. The Spearman’s rank correlation coefficient (r_s_) was used to assess construct validity. In order to further investigate validity of the construct, the correlation between the LSS and the single question about life satisfaction as a single item measure was calculated, as well.

To assess the test-retest reliability, relative and absolute reliability indices were calculated. The relative reliability was measured by the two way random effects model of intraclass correlation coefficient (ICC2,1) (Shrout and Fleiss 1979), and ICC ≥0.6 was considered satisfactory (Chinn 1991).The standard error of measurement (SEM) was calculated to measure the absolute reliability (Weir 2005). Also to detect any possible systematic bias, the paired sample t-test between test and retest mean scores was performed (Bruton et al. 2000).

### Ethics

The study was approved by the in Ethics Committee of the University of Social Welfare and Rehabilitation Sciences, Tehran, Iran. All participants were informed about the purpose of the study, and an informed consent was obtained from each participant. The LSS was used in this research, with the developers’ permission.

## Results

### Descriptive Statistics

334 cognitively healthy elderly (mean age = 65.61, Range = 60–86), participated in the present study. Sixty percent of them were male. Fifty three percent of them had high school educational level, and others had a university degree (Table 1).

**Table 1:** Demographic characteristics of participants (*n* = 334)

Variable	n	Percent
Gender		
Female	200	60
Male	134	40
Education		
Less than 12 years and high school graduate	284	85
University degree	50	15

According to the results of the Kolmogorov-Smirnov test, the distribution of the items’ scores of the LSS was not normal (*p* < 0.05), therefore the PLS path modeling can be used.

### The Measurement Model

We test the measurement model to ascertain the overall psychometric properties of the scales. Indicator reliability was determined by examining construct item loading. All items had loadings above 0.50. The scale had good internal consistency as indexed by the Cronbach’s α and composite reliability. In addition, the average variance extracted (AVE) for each measure exceeded 0.50.

Convergent validity analysis results are represented in Table 2.

**Table 2:** Convergent validity analysis (n = 334)

Construct	Composite reliability	Cronbach’s α	AVE
Daily activity	0.86	0.79	0.56
Meaning	0.88	0.82	0.59
Goals	0.83	0.73	0.50
Mood	0.88	0.82	0.58
Self-concept	0.83	0.76	0.52
Health	0.89	0.84	0.61
Finances	0.91	0.87	0.67
Social contacts	0.84	0.73	0.54
LSS	0.95	0.95	

Comparison of item loadings with item cross loadings was used for discriminant validity. All items loaded more highly on their respective constructs than other constructs. Therefore the constructs demonstrate adequate discriminant validity (Table 3).

**Table 3:** Item loadings and cross loadings for 40 items of the LSS (n = 334)

Item				Construct				
	Daily Activity	Finances	Goals	Health	Meaning	Mood	Self-concept	Social contacts
DA1	**0.780**	0.436	0.583	0.282	0.714	0.611	0.592	0.406
DA2	**0.513**	0.315	0.354	0.263	0.286	0.350	0.298	0.338
DA3	**0.764**	0.423	0.539	0.271	0.546	0.485	0.499	0.312
DA4	**0.859**	0.488	0.638	0.285	0.678	0.587	0.610	0.517
DA5	**0.770**	0.380	0.528	0.323	0.631	0.582	0.497	0.440
F1	0.453	**0.848**	0.522	0.242	0.530	0.395	0.487	0.280
F2	0.462	**0.874**	0.555	0.248	0.541	0.338	0.488	0.273
F3	0.468	**0.814**	0.466	0.194	0.553	0.361	0.416	0.313
F4	0.495	**0.867**	0.565	0.227	0.558	0.394	0.489	0.322
F5	0.366	**0.669**	0.476	0.197	0.424	0.342	0.330	0.331
G1	0.310	0.149	**0.493**	0.144	0.330	0.274	0.345	0.298
G2	0.509	0.516	**0.799**	0.256	0.548	0.431	0.534	0.443
G3	0.556	0.525	**0.773**	0.309	0.589	0.423	0.498	0.380
G4	0.563	0.427	**0.704**	0.273	0.548	0.423	0.518	0.384
G5	0.535	0.506	**0.689**	0.184	0.629	0.480	0.456	0.398
H1	0.294	0.216	0.287	**0.838**	0.331	0.340	0.263	0.146
H2	0.232	0.122	0.198	**0.760**	0.167	0.209	0.137	0.121
H3	0.270	0.215	0.245	**0.710**	0.267	0.275	0.217	0.191
H4	0.401	0.278	0.354	**0.865**	0.415	0.462	0.357	0.250
H5	0.220	0.188	0.184	**0.711**	0.190	0.213	0.113	0.134
Me1	0.575	0.468	0.588	0.262	**0.775**	0.541	0.529	0.363
Me2	0.644	0.490	0.625	0.278	**0.795**	0.580	0.657	0.471
Me3	0.540	0.491	0.465	0.367	**0.709**	0.537	0.437	0.239
Me4	0.648	0.446	0.647	0.287	**0.734**	0.509	0.539	0.366
Me5	0.606	0.555	0.609	0.248	**0.808**	0.587	0.542	0.381
M1	0.546	0.302	0.450	0.316	0.609	**0.757**	0.521	0.444
M2	0.645	0.466	0.541	0.372	0.640	**0.847**	0.574	0.466
M3	0.543	0.320	0.449	0.319	0.489	**0.771**	0.485	0.499
M4	0.403	0.238	0.320	0.187	0.374	**0.663**	0.336	0.392
M5	0.537	0.340	0.445	0.327	0.587	**0.752**	0.497	0.349
Sec1	0.635	0.498	0.630	0.376	0.690	0.591	**0.835**	0.426
Sec2	0.462	0.421	0.484	0.062	0.461	0.416	**0.756**	0.414
Sec3	0.599	0.441	0.550	0.341	0.621	0.580	**0.820**	0.402
Sec4	0.228	0.170	0.240	0.203	0.228	0.298	**0.364**	0.242
Sec5	0.431	0.349	0.446	0.039	0.430	0.364	**0.731**	0.372
SO1	0.383	0.268	0.451	0.152	0.395	0.432	0.428	**0.842**
SO2	0.364	0.296	0.309	0.129	0.257	0.365	0.362	**0.582**
SO3	0.408	0.288	0.477	0.154	0.411	0.439	0.441	**0.867**
SO4	0.371	0.218	0.319	0.099	0.273	0.380	0.278	**0.656**
SO5	0.459	0.279	0.411	0.275	0.392	0.443	0.372	**0.672**

DA, Daily Activity; Me, Meaning; G, Goals; M, Mood; Sec, Self-concept; H, Health; F, Finances and SO, Social Contacts from the LSS

The boldface indicate items' loding on their respective constructs

In addition, Fornell-Larcker Criterion Analysis for Checking Discriminant Validity was done (Table 4). AVE is written in bold on the diagonal of the table. These diagonal elements are greater than their corresponding correlation coefficients except the AVE for the construct Goal and the correlation between the constructs Goal and Daily activity.

**Table 4:**
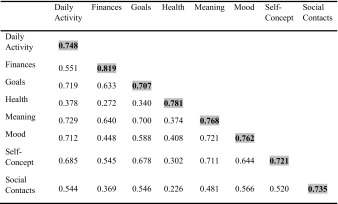
Fornell-Larcker criterion analysis for checking discriminant validity (n = 334)

### The Structural Model

**Fig. 1:**
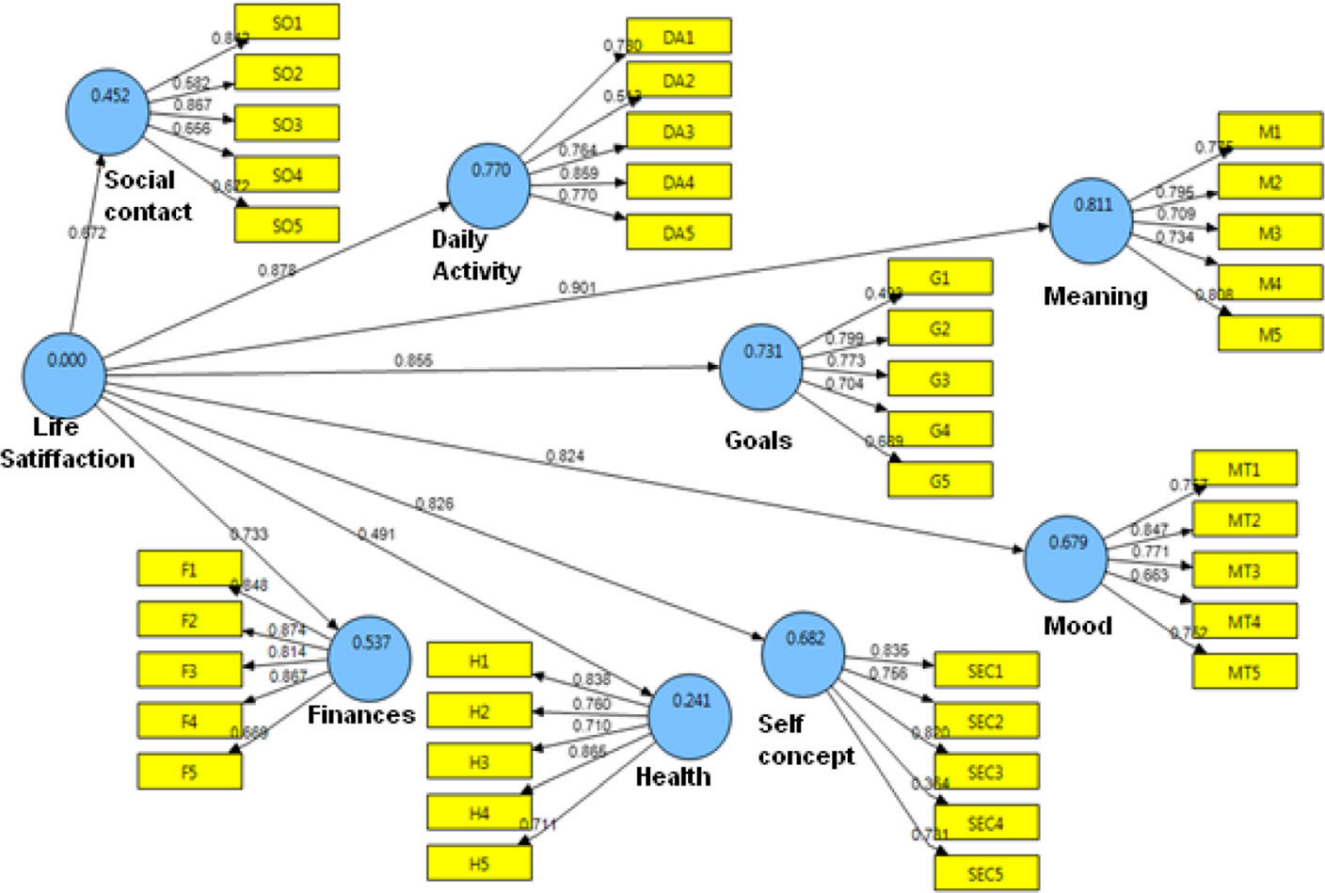
Structural model results of the LSS

Figure 1 shows the structural model results. The path between life satisfaction and all eight factors were highly significant. All beta coefficients are positive and statistically significant (*p* < 0.001).

Coefficients of determination (R-squares) are represented in Table 5. A considerable percent of the factors was accounted by their explanatory construct; life satisfaction.

**Table 5:** Coefficients of determination for factors (n = 334)

	Daily Activity	Finances	Goals	Health	Meaning	Mood	Self-concept	Social contacts
R Square	0. 77	0.54	0.73	0.24	0.81	0. 68	0.68	0.45

The goodness-of-fit index (GoF) value was 0.59 witch could be interpreted as an acceptable value (Wetzels et al. 2009).

### Further Evidences of the Construct Validity and Test-Retest Reliability

The correlation between the LSS mood scores and SF-36 emotional wellbeing scores was 0.65 and it was the highest value among all correlation coefficients between SF-36 emotional wellbeing subscale and all other subscales of the LSS. In addition, the correlation between the LSS health scores and SF-36 general health perception scores was 0.55 and it was the highest value among all correlation coefficients between SF-36 general health perception subscale and all other subscales of LSS. These results confirm the validity of the mentioned subscales (Table 6).

**Table 6:** Spearman’s rank correlation coefficient of the LSS and SF-36 subscales (n = 334)

LSS SF36	DA	Me	G	M	Sec	H	F	SO
PF	.267^**^	.229^**^	.198^**^	.200^**^	.213^**^	.542^**^	.200^**^	.117
RLP	.285^**^	.318^**^	.256^**^	.241^**^	.177^**^	.405^**^	.241^**^	.139^*^
RLE	.328^**^	.349^**^	.284^**^	.278^**^	.242^**^	.231^**^	.255^**^	.149^*^
EF	.538^**^	.483^**^	.388^**^	.588^**^	.460^**^	.498^**^	.345^**^	.379^**^
EW	.575^**^	.558^**^	.443^**^	.653^**^	.472^**^	.346^**^	.376^**^	.476^**^
SF	.382^**^	.342^**^	.246^**^	.339^**^	.230^**^	.331^**^	.219^**^	.212^**^
P	.338^**^	.345^**^	.266^**^	.308^**^	.190^**^	.572^**^	.272^**^	.149^*^
GH	.482^**^	.467^**^	.373^**^	.423^**^	.432^**^	.555^**^	.353^**^	.296^**^

DA: Daily Activity; Me: Meaning; G: Goals; M: Mood; Sec: Self-concept; H: Health; F: Finances and SO: Social Contacts from the LSS. PF: Physical Functioning; RLP: Role Limitations due to Physical Health Problems; RLE: Role Limitations due to Emotional Problems; EW: Emotional Well-being; SF: Social Functioning; EF: Energy/Fatigue; P: Bodily Pain and GH: General Health Perceptions from the SF-36

*P < 0.05

**P < 0.01

Also, the correlation between LSS and the single question about life satisfaction asked from participants was 0.60 (*p* < .001).

Table 7 represents the reliability measures including test-retest reliability measures (ICC and SEM) for each subscales and the total score of LSS. The Intraclass correlation coefficient (ICC) was 0.91 for the overall scale, and 0.76–0.96 for the subscales.

**Table 7:** Test-retest reliability of the LSS subscales (*n* = 50)

	Test mean (SD)	Median	Retest mean (SD)	ICC (95% CI)	S.E.M
Daily activity	17.04 (3.01)	17.5	17.37 (2.68)	.76 (.57 .86)	1.92
Meaning	17.14 (3.11)	17.7	17.17 (2.64)	.86 (.75 .92)	1.44
Goals	16.73 (2.78)	17.00	17.31 (2.30)	.83 (.70 .90)	1.34
Mood	18.62 (3.00)	19.00	18.39 (2.91)	.85 (.73 .91)	1.66
Self-Concept	17.90 (2.27)	18.00	17.55 (2.21)	.92 (.87 .96)	.81
Health	15.73 (3.78)	16.00	15.92 (4.11)	.96 (.93 .98)	1.07
Finances	16.09 (3.83)	16.00	16.45 (3.83)	.95 (.91 .97)	1.20
Social contacts	18.62 (2.72)	16.00	18.65 (2.64)	.86 (.75 .92)	1.30
LSS	137.92 (18.53)	139	138.84 (17.78)	.91 (.84 .95)	7.73

S.E.M, Standard Error of Measurement; ICC, Intraclass Correlation Coefficient

The results of paired sample t-test indicated that the questionnaire scores were not significantly different (*P* ≥ .05) in the two administrations of test and retest.

## Discussion

The purpose of the present study was to translate the “Life satisfaction Scale (LSS)” and evaluate psychometric properties of the Persian version of this scale. The translation of the LSS followed a standard protocol developed by International Quality of Life Assessment (IQOLA), including multiple forward and backward translations, assessment of quality of translation and evaluating its conceptual equivalence with the original version (Ware 2004). The LSS consists of 40 short and clear items and response choices. In the translation process of the present study, the translators did not encounter any obscure or challenging item for translation. After producing and documenting individual translations, consensus was reached among the translation team and they agreed on a common translation. In addition, during the cognitive debriefing interview, we didn’t find any obscure, confusing and difficult items. As the LSS was a well-designed scale, we could achieve a good Persian version of this scale.

In the present study, the psychometric properties of the Persian version of the LSS were evaluated. Construct validity of the LSS was supported by satisfactory convergent and discriminant validity of the constructs. The LSS demonstrate adequate discriminant validity based on the comparison of item loadings with item cross loadings and Fornell and Larcker’s test results, considering only one exception in the case of the near values of the AVE for the construct Goal and the correlation between the constructs Goal and Daily activity.

Reliability is a major concern when we use a psychological test. The results of the present study indicate that the measures are robust in terms of their internal consistency as indexed by the Cronbach’s α (ranges from .73–.87 for each subscale, and .93 for the LSS total scores) and the composite reliability. These results are well fit with the results of the LSS original version (the Cronbach’s α ranges from .60 to .79 for each subscale, and .93 for the LSS total score) (Salamon and Conte 2003).

In the current study, Item’s factor loading results demonstrated that in some instances, items loads significantly on more than one construct. The item DA1 loads similarly on its corresponding construct i.e. Daily Activity (loading = .780) and on the other construct i.e. Meaning (loading = .714); Also, item G5 loads on Goals (loading = .689) and on Meaning (loading = .629). In other words, the items have cross loaded on the construct “Meaning” as well as their related constructs. One possible explanation for these findings is that in Persian language, the concepts “Function”, “Goals” and “Meaning” are interrelated concepts. The item DA1 exactly refers to satisfaction with individual’s daily routine. The satisfaction with individual’s daily routine is dependent on his feeling that his or her life is meaningful or not. Similarly, the item G5 refers to satisfaction with the way things turn out. This item belongs to the Goals construct. However, the item may relate to the meaning of life (Meaning construct) as well. Therefore, the nature of these items may lead to their high loadings on more than one construct. Nevertheless, the loading of each item on its corresponding construct is higher than its loading on the other construct. Therefore, the results confirmed the structural model of the LSS.

On the other hand, similar results were reported by Salamon and Conte (2003) in the Procrustes confirmatory factor analysis of original version of the LSS. Their results indicated that two constructs (the Health and the Finance) show complete congruence with their corresponding items. The other six factors produced a number of significant loadings and in some instances, variables loads significantly on more than one construct (Salamon and Conte 2003).

Moreover, the loading of SC4 on its corresponding construct was 0.364 (t value exceeds 1.96) in the present study. More analysis demonstrated that eliminating the item from the model did not improve the model fit. On the other hand, the item’s loading value was close to 0.4 and consistent with the original version (loading = 0.404). Therefore, we decided not to eliminate SC4 from the model.

The results of the test-retest reliability study might be considered as an evidence of good scale’s stability over time. In the present study, good test-retest reliability of Persian version of LSS (ICC = 0.84) was obtained. The results were consistent with original version of the LSS (the test-retest correlation coefficient of 0.67) (Salamon and Conte 2003).

Construct validity was supported by the presence of higher correlations between the LSS and the SF-36 subscale measuring similar constructs, and lower correlations between the subscales measuring dissimilar constructs. The correlation between the LSS and the single question about life satisfaction, as a single item measure, confirmed the validity of the questionnaire.

In order to assess the eight-factor structural model, the overall model goodness of fit has been determined through paths, coefficients of determination and the global criterion of goodness-of-fit (GoF). The results indicated an adequate goodness of fit between the hypothesized model of the LSS and the model derived from the sample data. It is notable that the fit measures of Covariance Based Structural Equation Modeling (CB-SEM) can be better suited for model validation compared to PLS’GoF (Henseler and Sarstedt 2013). However, in the present study we preferred to use PLS path modeling as the distribution of the data was not normal (Bagozzi 1994a, b; Hair et al. 2011). In addition, there are some studies that show PLS path modeling is appropriate for the confirmatory factor analysis which is more reliable and valid compared to Covariance Based Structural Equation Modeling (CB-SEM) (Hair et al. 2011; Afthanorhan 2013).

The “Life Satisfaction Scale” has been used as an outcome measure, measure of clinical need, and as a part of research protocols in a variety of settings. Numerous studies have confirmed its optimum psychometric properties (Salamon and Conte 2003). The results of the present study demonstrated that the Persian version of the LSS has good psychometric properties (internal consistency, test re-test reliability, and construct validity) to be used as an outcome measure in Iranian older adults.

The limitation is that the eligibility criteria for participation in this study were older adult’s literacy level (at least 9 years of school education) which may limit the generalization of its usefulness to a group of illiterate older adults. Furthermore, qualitative studies are suggested to find the meaning of life satisfaction of the Iranian elderly population in more depth.

## Conclusion

In conclusion, the Persian version of the Life Satisfaction Scale (LSS) is a reliable and valid instrument for measuring life satisfaction in the Iranian older adults.

## References

[CR1] Afthanorhan W (2013). A comparison of partial least square structural equation modeling (PLS-SEM) and covariance based structural equation modeling (CB-SEM) for confirmatory factor analysis. International Journal of Engineering Science and Innovative Technology.

[CR2] Amini R, Ingman SR, Sahaf R (2013). Aging in iran: Past, present and future. The Journal of Aging in Emerging Economies.

[CR3] Aprahamian I, Martinelli JE, Neri AL, Yassuda MS (2009). The clock drawing test. A review of its accuracy in screening for dementia. Dementia & Neuropsychologia.

[CR4] Bagozzi RP (1994). Advanced methods of marketing research.

[CR5] Bagozzi RP, Bagozzi RP (1994). Advanced topics in structural equation models. Advanced methods of marketing research.

[CR6] Barclay D, Higgins C, Thompson R (1995). The partial least squares (PLS) approach to causal modeling: Personal computer adoption and use as an illustration. Technology Studies.

[CR7] Bloom DE, Boersch-Supan A, McGee P, Seike A (2011). Population aging: Facts, challenges, and responses. Benefits and Compensation International.

[CR8] Brodaty H, Moore CM (1997). The clock drawing test for dementia of the Alzheimer's type: A comparison of three scoring methods in a memory disorders clinic. International Journal of Geriatric Psychiatry.

[CR9] Bruton A, Conway JH, Holgate ST (2000). Reliability: What is it, and how is it measured?. Physiotherapy.

[CR10] Bullinger M, Alonso J, Apolone G, Leplège A, Sullivan M, Wood-Dauphinee S, Gandek B, Wagner A, Aaronson N, Bech P (1998). Translating health status questionnaires and evaluating their quality: The IQOLA project approach. Journal of Clinical Epidemiology.

[CR11] Byrne BM (2001). Structural equation modeling with AMOS, EQS, and LISREL: Comparative approaches to testing for the factorial validity of a measuring instrument. International Journal of Testing.

[CR12] Cahn DA, Salmon DP, Monsch AU e a (1996). Screening for dementia of the Alzheimer type in the community: The utility of the clock drawing test. Archives of Clinical Neuropsychology.

[CR13] Chinn S (1991). Statistics in respiratory medicine. 2. Repeatability and method comparison. Thorax.

[CR14] Comrey A, Lee H (1992). A first course in factor analysis.

[CR15] Fernandez-Ballesteros R, Dolores Zamaron M, Angel Ruiz M (2001). The contribution of socio-demographic and psychosocial factors to life satisfaction. Ageing and Society.

[CR16] Fornell C (1982). A second generation of multivariate analysis. 2. Measurement and evaluation.

[CR17] Fornell C, Larcker DF (1981). Evaluating structural equation models with unobservable variables and measurement error. Journal of Marketing Research.

[CR18] Gefen D, Straub D (2005). A practical guide to factorial validity using PLS-graph: Tutorial and annotated example. Communications of the Association for Information Systems.

[CR19] Gefen D, Straub D, Boudreau M-C (2000). Structural equation modeling and regression: Guidelines for research practice. Communications of the Association for Information Systems.

[CR20] Gil-Garcia, J. R. (2008). Using partial least squares in digital government research. *Handbook of Research on Public Information Technology. Hershey: Idea Group Inc pp*, 239–253. 10.4018/978-1-59904-857-4.ch023.

[CR21] Hair JF, Ringle CM, Sarstedt M (2011). PLS-SEM: Indeed a silver bullet. Journal of Marketing Theory and Practice.

[CR22] Henseler J, Sarstedt M (2013). Goodness-of-fit indices for partial least squares path modeling. Computational Statistics.

[CR23] Hubbard EJ, Santini V, Blankevoort CG e a (2008). Clock drawing performance in cognitively normal elderly. Archives of Clinical Neuropsychology.

[CR24] Hulland J, Business RIS o (1999). Use of partial least squares (PLS) in strategic management research: A review of four recent studies. Strategic Management Journal.

[CR25] Kline RB (2011). Principles and practice of structural equation modeling.

[CR26] Lux, T. & Scherger, S. (2017). By the sweat of their brow? The effects of starting work again after pension age on life satisfaction in Germany and the United Kingdom. *Ageing and Society, 37*(2), 295–324.

[CR27] Mehryar, A. H., & Ahmad-Nia, S. (2004). Age-structural transition in Iran: Short and long-term consequences of drastic fertility swings during the final decades of twentieth century. Age-structural transitions: Population waves, disordered cohort flows and the demographic bonus, Paris, 23–26 February 2004.

[CR28] Montazeri A, Goshtasebi A, Vahdaninia M, Gandek B (2005). The short form health survey (SF-36): Translation and validation study of the Iranian version. Quality of Life Research.

[CR29] Nair, A. K., Gavette, B., Damman, M., Dekker, W., Green, R. C., Mandel, A., et al. (2010). Clock drawing test ratings by dementia specialists: Interrater reliability and diagnostic accuracy. *The Journal of Neuropsychiatry and Clinical Neurosciences, 22*(1), 85–92. 10.1176/appi.neuropsych.22.1.85 PMC2938787 20160214

[CR30] Nunnally JC, Bernstein IH, Berge JM t (1967). Psychometric theory.

[CR31] Parker PD, Martin AJ, Marsh HW (2008). Factors predicting life satisfaction: A process model of personality, multidimensional self-concept, and life satisfaction. Australian Journal of Guidance and Counselling.

[CR32] Pavot W, Diener E, Colvin CR, Sandvik E (1991). Further validation of the satisfaction with life scale: Evidence for the cross-method convergence of well-being measures. Journal of Personality Assessment.

[CR33] Rosipal R, Krämer N (2006). Overview and recent advances in partial least squares. Subspace, latent structure and feature selection.

[CR34] Salamon MJ (1988). Clinical use of the life satisfaction in the elderly scale. Clinical Gerontologist.

[CR35] Salamon M, Conte V (2003). Manual for the life satisfaction scale (LSS): Formerly the life satisfaction in the elderly scale (LSES).

[CR36] Shirazikhah M, Mousavi MT, Sahaf R, Sarmadi M (2012). Consequence of changes in the elderly people population: elderly women in Iran. Life Science Journal.

[CR37] Shrout PE, Fleiss JL (1979). Intraclass correlations: Uses in assessing rater reliability. Psychological Bulletin.

[CR38] Statistical Center of Iran (2011). Statistics, Census of Population and Housing of the year 2011. from http://www.amar.org.ir/Default.aspx?tabid=133.

[CR39] Tenenhaus M, Vinzi VE, Chatelin Y-M, Lauro C (2005). PLS path modeling. Computational statistics & data analysis.

[CR40] Terwee CB, Bot SD, de Boer MR e a (2007). Quality criteria were proposed for measurement properties of health status questionnaires. Journal of Clinical Epidemiology.

[CR41] Veenhoven, R. (1996). The study of life satisfaction. In W. E. Saris, R. Veenhoven, A.C. Scherpenzeel, & B. Bunting (Eds.) *A comparative study of satisfaction with life in Europe* (pp. 11–48). Budapest: Eötvös University Press.

[CR42] Ware JE (2004). SF-36 health survey update. The use of psychological testing for treatment planning and outcomes assessment.

[CR43] Ware Jr JE, Sherbourne CD (1992). The MOS 36-item short-form health survey (SF-36): I. Conceptual framework and item selection. Medical Care.

[CR44] Weir JP (2005). Quantifying test-retest reliability using the intraclass correlation coefficient and the SEM. The Journal of Strength & Conditioning Research.

[CR45] Wetzels M, Odekerken-Schröder G, Van Oppen C (2009). Using PLS path modeling for assessing hierarchical construct models: Guidelines and empirical illustration. MIS Quarterly.

[CR46] Yu S-C, Hsu W-H (2012). Applying structural equation modeling methodology to test validation: An example of cyberspace positive psychology scale. Quality & Quantity.

